# Reversal of hepatic encephalopathy following a trans-scrotal approach to mesogonadal shunt embolization: a case report

**DOI:** 10.1186/s42155-026-00726-3

**Published:** 2026-07-06

**Authors:** Adil Basha, Ifeadikanwa Emejulu, Swar Shah

**Affiliations:** 1 Texas A&M University Health Science Center, Bryan, TX USA; 2 Department of Interventional Radiology, Baylor University Medical Center, Dallas, TX 75246 USA

**Keywords:** Hepatic encephalopathy, Embolization, Portosystemic shunt, Cirrhosis, Portal hypertension

## Abstract

**Background:**

Spontaneous portosystemic shunts are defined as venous conduits that occur in response to elevated portal venous pressure, resulting in alternative outflows from the portal circulation. We present a case of atypical portosystemic shunt formation between the inferior mesenteric vein branches and the left testicular vein resulting in hepatic encephalopathy in a patient with a history of cirrhosis, describing the access technique we used for successful portosystemic shunt embolization via a combined right common femoral vein and trans-scrotal approach.

**Case presentation:**

A 57-year-old male with a history of cirrhosis presented with a 1-month history of progressive cognitive decline despite adherence to a medical regimen of lactulose, rifaximin, and a low-sodium diet. Abdominal computerized tomography and scrotal ultrasound revealed shunt formation from the mesenteric venous system to the systemic circulation, with intermediate connections through the scrotal venous plexus and left gonadal vein. Following an outpatient consultation with interventional radiology for worsening hepatic encephalopathy, the inflow and outflow tracts of the shunt were visualized using digital-subtraction venography. Embolization with Penumbra Ruby XL coil packs and Sotradecol infusion was achieved using an ultrasound-guided trans-scrotal approach for the inflow tract and a femoral approach for the outflow tract. The patient’s hepatic encephalopathy resolved shortly after the procedure.

**Conclusions:**

Mesogonadal shunts can be a sequela to portal hypertension and leave patients susceptible to hepatic encephalopathy due to direct entry of metabolic toxins such as ammonia into the systemic circulation. Although systemic access is the standard approach for existing coil embolization and balloon-occluded retrograde transvenous obliteration (BRTO) procedures used for the treatment of mesogonadal shunts, further consideration should be given for unconventional vascular access in difficult portosystemic shunt presentations to better address shunt inflow and prevent recurrence. Future avenues for research can include comparing shunt recurrences in patients with layered sclerosant and coil embolization to patients with BRTO or coil embolization alone.

## Background

Spontaneous portosystemic shunts (SPSS) are defined as venous conduits that occur in response to elevated portal venous pressure, typically in the context of cirrhotic liver disease [1]. SPSS can result in alternative outflows from the portal circulation, allowing blood to enter the systemic venous circulation while bypassing the liver, limiting ammonia metabolism and promoting the development of hepatic encephalopathy [2].

## Case presentation

### History and physical examination

A 57-year-old male with a history of human immunodeficiency virus (HIV) on antiretroviral therapy, cirrhosis secondary to MASH, type 2 diabetes, and hypertension presented with a 1-month history of progressive cognitive decline despite adherence to a medical regimen of lactulose, rifaximin, and a low-sodium diet. He was seen for an outpatient consultation with Interventional Radiology in October 2025 for recurrent encephalopathy, which appeared to be rapidly developing, characterized by episodic confusion, falls, and word finding difficulties. While an intervention was planned, he was admitted to the hospital less than 2 weeks later with notable dysarthria and a diminished expressive vocabulary.

He had a previous history of esophageal varices secondary to portal hypertension which was managed with endoscopic band ligation in March 2025, as well as a large splenorenal shunt which was embolized with coils in 2023 for encephalopathy. His notable surgical history included bilateral inguinal hernia repairs.

### Diagnostic evaluation

Laboratory investigations demonstrated hyperammonemia (135 µmol/L), hyperbilirubinemia (2.9 mg/dL), hypoalbuminemia (3.3 g/dL), hyperglobulinemia (3.9 g/dL), leukopenia (3800/µL), and thrombocytopenia (40,000/µL), consistent with advanced chronic liver disease. His HIV was controlled with an undetectable viral load and CD4 count of 412/µL.

Initial screening for acute secondary causes—including chest radiography, head computerized tomography (CT), and esophagogastroduodenoscopy—was unremarkable. Recent outpatient screening for hepatocellular carcinoma (ultrasound and alpha-fetoprotein) was negative. A dedicated CT of the abdomen and pelvis performed 1 month prior demonstrated a complex portosystemic shunt arising from the sigmoid and left colic veins, extending into the left scrotum and draining via the left testicular vein into the systemic circulation.

After a discussion of the risks and benefits of proceeding with intervention, notably including potential colonic venous ischemia, testicular ischemia, nontarget embolization, and uncontrolled bleeding, both the patient and his proxy agreed with proceeding to embolization.

### Procedure

**Fig. 1:**
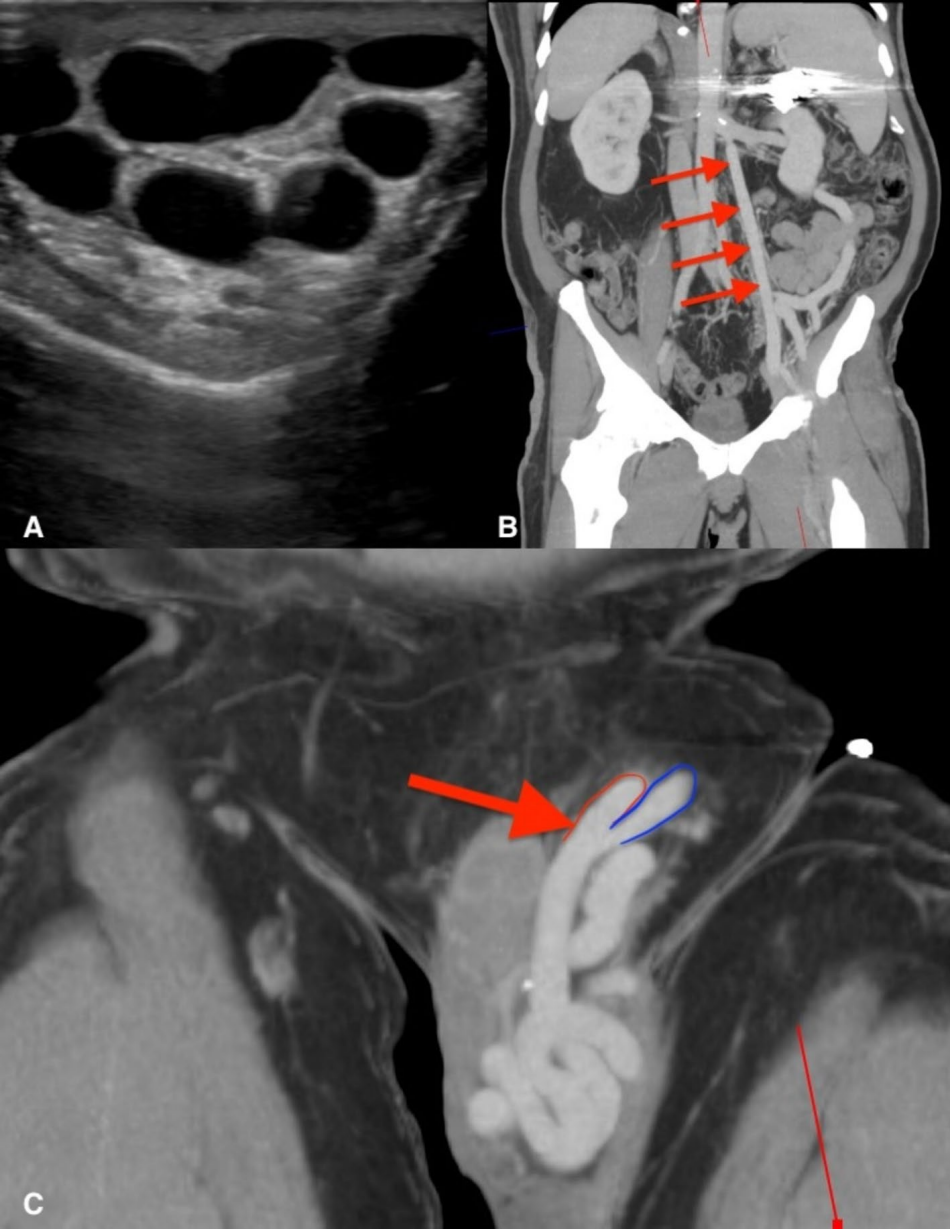
**A** A transabdominal ultrasound demonstrated complex scrotal venous anatomy. **B** On coronal view, a dilated left gonadal vein can be seen draining into the left renal vein (red arrows). **C** The confluence (red arrow) of the sigmoid and left colic vein branches (red and blue tracings) is seen within the scrotum with complex venous flow

Initial CT imaging of the abdomen and pelvis revealed a confluence of the sigmoid and colic vein branches forming the inflow tract of the shunt, as well as a dilated left gonadal vein feeding into the left renal vein. A splenorenal shunt that had been previously undergone coil embolization was also seen on the scan. Sonographic evaluation revealed complex scrotal venous architecture, with a dilated pampiniform plexus as would be seen in a varicocele (Fig. 1A). Within the left lower quadrant, two dominant afferent branches were identified originating from the descending and sigmoid colon, respectively unifying at the upper aspect of the scrotum. These vessels traversed the inguinal canal into the left scrotum, forming a high-flow venous plexus with efferent drainage via a dilated left testicular vein (Fig. 1B). This was consistent with prior CT findings (Fig. 1B, C). Given the anatomical complexity of this portosystemic shunt, a hybrid access strategy utilizing both systemic venous and direct trans-scrotal routes was selected to facilitate comprehensive management of the inflow and outflow components.

After systemic venous access was obtained through the right common femoral vein using a 4-French micropuncture set, the 0.018″ microwire was exchanged for a 0.035″ 15-cm Bentson wire. A 7-French sheath was placed in the left renal vein over the Bentson wire, which was exchanged for a 0.035″ 150-cm Rosen wire. A 5-French 100-cm Cobra catheter was placed over the Rosen wire and used to reach the testicular vein outflow tract. A 4-French 9 mm × 40 cm Edwards Fogarty balloon was then advanced into the lower aspect of the testicular vein, and a balloon-occluded retrograde venogram was performed, confirming a large, high-flow portosystemic shunt with outflow through the left testicular vein.

**Fig. 2:**
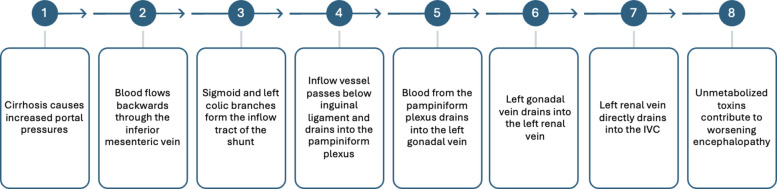
A schematic diagram detailing the flow of the mesogonadal shunt from the portal to systemic circulation

**Fig. 3:**
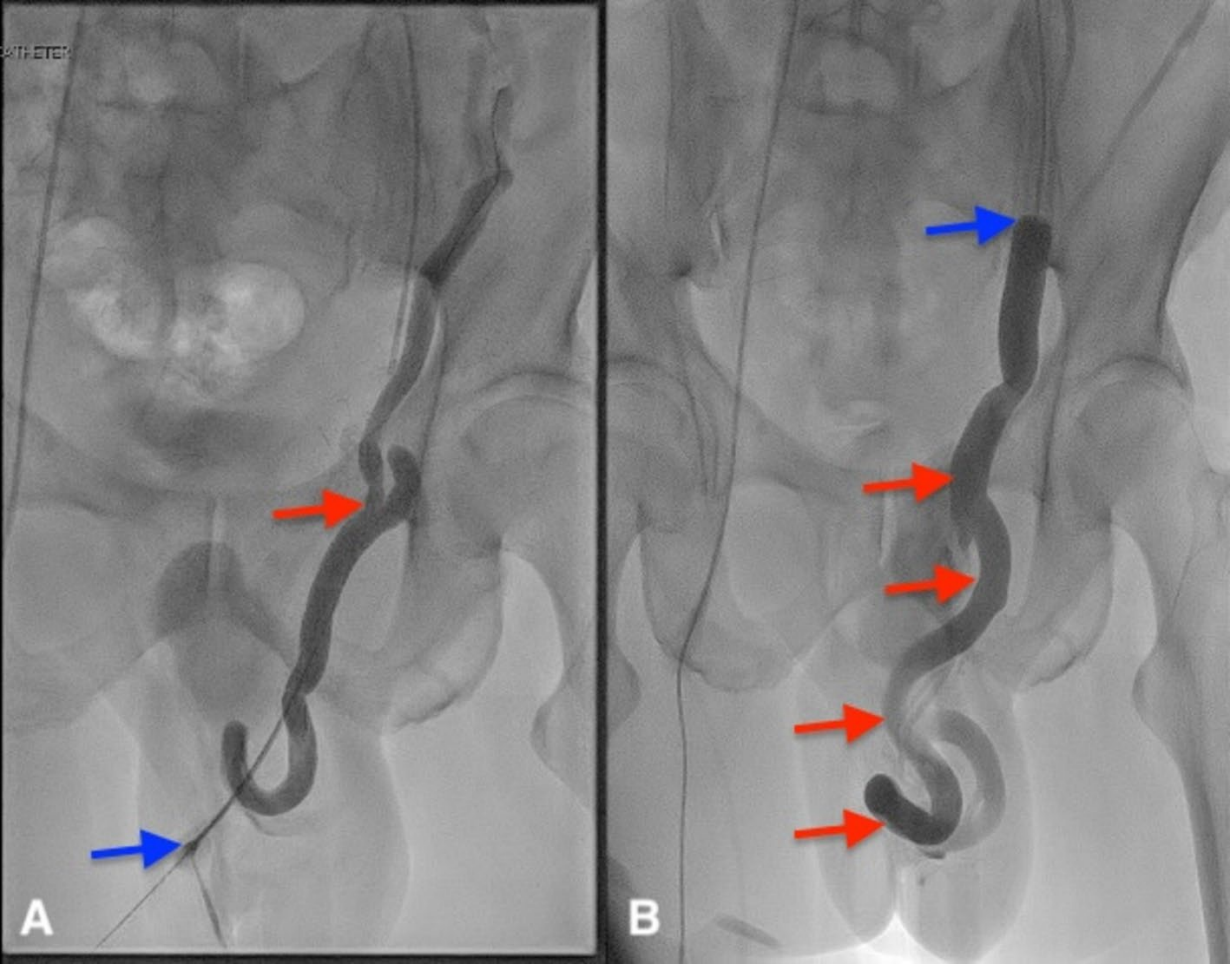
**A** A digital subtraction venogram was performed via trans-scrotal access (blue arrow), with contrast administration resulting in reflux which demonstrated the inflow branches from the sigmoid and left colic veins (red arrows). **B** A second venogram was performed via right common femoral vein access and Fogarty balloon inflation (blue arrow), demonstrating complex flow within the scrotum and outflow through the left testicular vein (red arrows)

After careful mapping with ultrasound, and under sterile sonographic guidance, trans-scrotal access of the unified inflow vein was performed using a 21-gauge micropuncture needle with placement of a 0.018″ microwire, followed by the insertion of a 4/5-French 10-cm GlideSheath Slender introducer sheath. A digital subtraction sheath venogram displayed the entirety of the shunt, with contrast reflux demonstrating inflow branches from the descending and sigmoid colon, complex flow through the scrotum and outflow via the left testicular vein (Figs. 2 and 3).

**Fig. 4:**
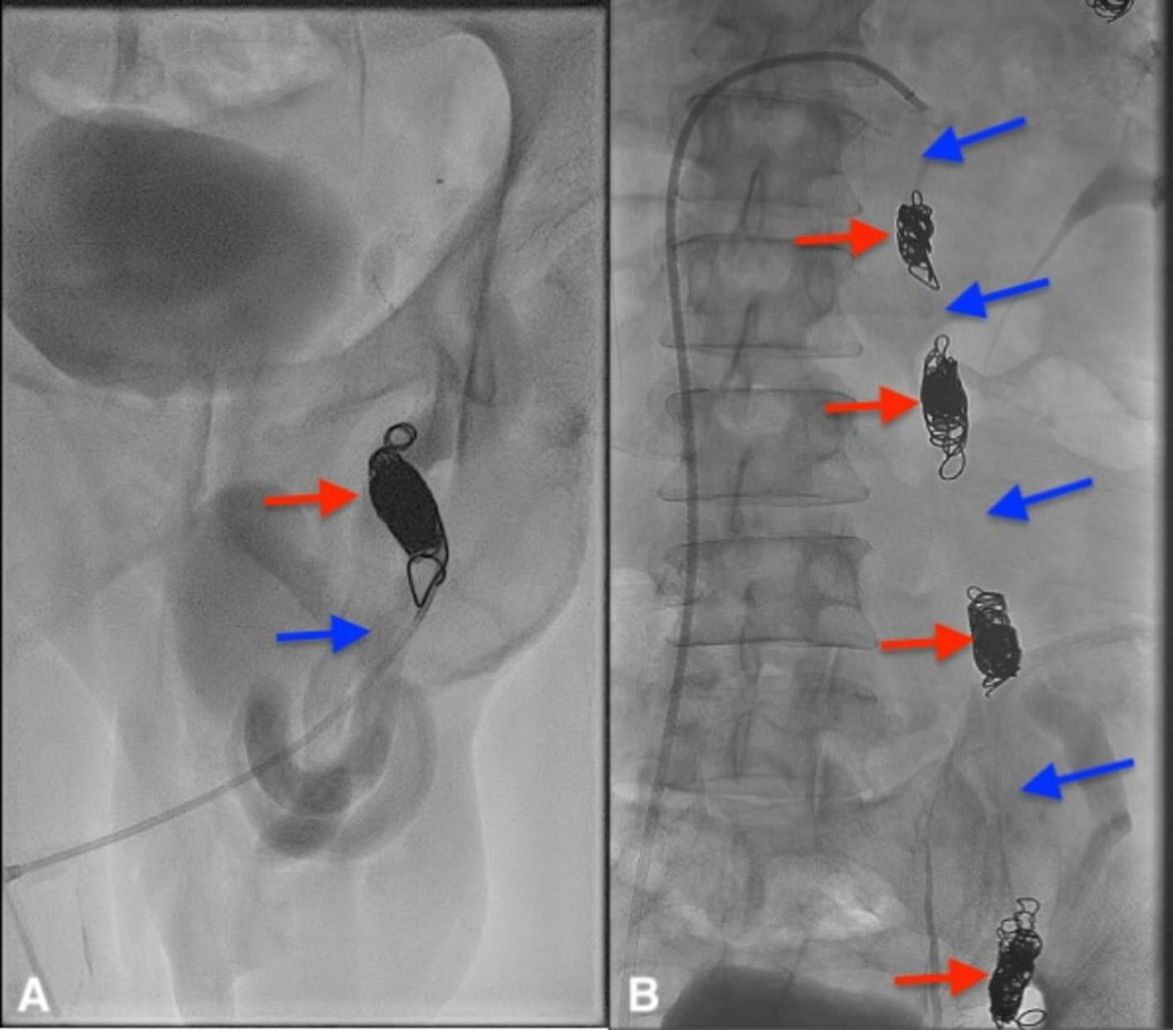
**A** The unified inflow vein was embolized below the confluence of the sigmoid branches and the left colic branches, and 1% Sotradecol was administered afterwards (blue arrow) to ensure adequate coverage and contact time with scrotal vasculature. **B** Four coil packs were placed along the outflow tract in a “sandwich” technique (red arrows), with 1% Sotradecol administered in between them, as shown by the gaps between the coils (blue arrows)

**Fig. 5:**
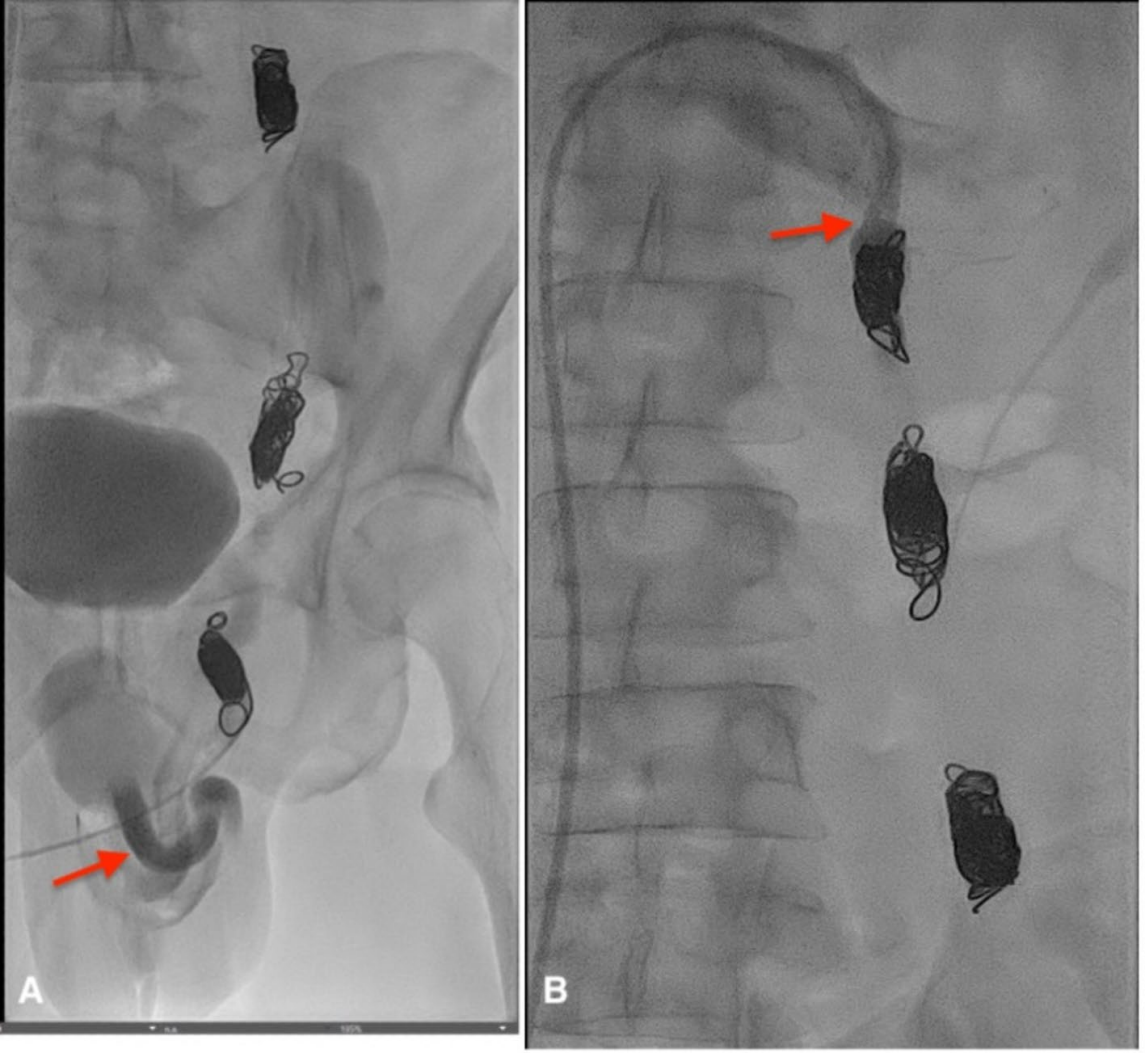
**A** Confirmatory trans-scrotal venogram displayed contrast stasis in the scrotal venous plexus (red arrow), confirming adequate inflow control. **B** Confirmatory trans-femoral venogram demonstrated no residual inflow (red arrow)

With the aid of a 5-French catheter, 0.030″ 70-cm Penumbra Ruby XL coils were used to embolize the unified inflow vein just below the confluence of the sigmoid branches and the left colic branches. Care was taken to avoid approaching these vessels as they appeared to represent the dominant outflow of the left colon and embolization would come with the risk of venous ischemia. A tight coil pack was successfully placed, followed by administration of 1% foamed Sotradecol via the same scrotal access under fluoroscopic monitoring to confirm adequate coverage and contact time with the scrotal vasculature (Fig. 4A). A confirmatory venogram displayed contrast stasis in the scrotal venous plexus, confirming adequate inflow control (Fig. 5A).

Subsequently, four separate Penumbra Ruby XL coil packs were placed with intervening 1% foamed Sotradecol placed along the course of the left testicular vein via systemic venous access in a “sandwich” technique to prevent collateral recanalization (Fig. 4B). The confirmatory left renal venogram demonstrated no residual inflow (Fig. 5B).

After confirming adequate stasis of flow, the trans-scrotal access was removed following Gelfoam tract embolization, and hemostasis was achieved with manual compression. The patient tolerated the procedure well and was returned to the inpatient service for observation, after which he was discharged 13 days later following improvement of his encephalopathy.

## Discussion

SPSS are very common in the cirrhotic patient population, with studies revealing that up to 60% of patients with liver disease show evidence of portosystemic shunts [3]. Some of the most common shunt types include splenorenal, gastrorenal and paraumbilical shunts [4–6]. Although these are considered to be the typical presentations of SPSS, atypical shunts causing hepatic encephalopathy have also been discussed in existing literature, including mesenteric-iliac shunts, portocaval shunts, and intrahepatic shunts [7–9].

The driving force behind SPSS formation lies in the large pressure gradient between cirrhotic portal venous pressure (12–20 mmHg) and caval pressure (2–6 mmHg). Subsequent blood flow from the portal system back into its tributaries (splenic, superior mesenteric and inferior mesenteric veins) exceeds venous capacitance in the smaller vessels, causing high flow pathways to form from pre-existing embryonal venous channels [10]. Under normal physiologic conditions, these channels are functionally collapsed and do not make significant contributions to venous return, but in the context of elevated portal pressures, Doppler studies have found high flow velocities in SPSS that exceed 30 cm/s, with shunt embolization causing further increases in portal pressures, implying that the shunts were diverting significant amounts of flow away from the liver [11].

A possible explanation for this patient’s atypical shunting may have been his history of splenorenal shunt embolization, resulting in portal blood flow seeking another low resistance pathway and subsequent development of the mesenteric-gonadal shunt, with complex scrotal venous architecture found in his imaging indicating potential dilation of his pampiniform plexus to accommodate for high levels of flow to the left gonadal vein and ultimately systemic circulation. Post-surgical changes from his prior hernia repair may have also played a role.

Portosystemic shunts have historically been addressed with balloon-occluded retrograde transvenous obliteration (BRTO) or standalone coil embolization to block off high-flow vessels [12]. As part of our treatment, a trans-scrotal approach followed by a combination of coil embolization and Sotradecol sclerosant infusion was done to appropriately control the inflow into the portosystemic shunt and reduce the risk for reformation of collaterals. For outflow control, an alternating coil embolization and sclerosant infusion technique was done to extensively cover a significant segment of the left gonadal vein. We chose this approach in order to prevent recanalization of the outflow tract or the creation of a direct shunt via neoangiogenesis between the inferior mesenteric and left gonadal veins due to their close anatomical proximity and multiple small collateral pathways known to drain into the left gonadal vein [13]. Although there are existing reports of mesogonadal shunts being treated with coil embolization, a literature search did not reveal any other written reports of a mesogonadal shunt being treated with trans-scrotal access, making this case report the first of its kind [14].

Although there is a lack of evidence of colonic injury secondary to Sotradecol sclerosant infusion, technical precautions were still taken due to the proximity of the inflow tract to the descending and sigmoid colon, with no signs of complications following the procedure [15, 16]. Historically, the stomach has tolerated Sotradecol well, both via a transvenous and endoscopic injection perspective; however, the inherent muscular and vascular structure of the stomach is absent from the colon.

An additional area for consideration included risk of portosystemic shunt recurrence due to the persistence of underlying increased portal pressure following the mesogonadal shunt closure. As this patient was not a candidate for transjugular intrahepatic portosystemic shunt (TIPS) given his history of hepatic encephalopathy, definitive treatment would necessitate liver transplant, which would be unfeasible in the short term and limited by his HIV status. As such, the mainstays of this patient’s treatment would primarily involve medical optimization with lactulose and rifaximin, and expectant management of new portosystemic shunts with periodic embolization.

## Conclusions

SPSS is a common consequence of portal hypertension, and leaves patients susceptible to hepatic encephalopathy due to direct entry of metabolic toxins such as ammonia into the systemic circulation. Mesogonadal shunts are one such type of atypical SPSS which can occur due to abnormal communication between the inferior mesenteric vein branches and the left gonadal vein. Although systemic access is the standard approach for existing coil embolization and BRTO procedures used for the treatment of mesogonadal shunts, further consideration should be given for unconventional vascular access in difficult SPSS presentations to better address shunt inflow and prevent recurrence. Future avenues for research can include comparing shunt recurrences in patients with layered Sotradecol infusion and coil embolization to patients with BRTO or coil embolization alone.

## Data Availability

Data sharing is not applicable to this article as no datasets were generated or analysed during the current study.

## Abbreviations

SPSS: Spontaneous portosystemic shunts
MASH: Metabolic dysfunction-associated steatohepatitis
HIV: Human immunodeficiency virus
CT: Computerized tomography
BRTO: Balloon-occluded retrograde transvenous obliteration
TIPS: Transjugular intrahepatic portosystemic shunt
